# Treatment of Peritonitis

**Published:** 1890-07

**Authors:** 


Treatment of Peritonitis.—Dr. W. E. B. Davis states (*New Orleans Medical and Surgical Journal*) that, from a study of local and general peritonitis, he concludes that the following indications for treatment must be arrived at: 1°. Promote absorption of the inflammatory products of simple peritonitis as rapidly as possible, and thus relieve the inflammation and prevent the possibility of septic peritonitis. 2°. In the early stage of peritonitis, whether simple or septic, where the cause cannot be determined, hasten the absorption of inflammatory products, etc., with purgatives. 3°. When medical treatment fails to give relief, septic fluids should be removed by operative procedure. 4°. In localized peritonitis — with circumscribed pus formation — the pus should be removed and the abscess cavity drained. 5°. In acute septic peritonitis operative procedure must be adopted early or there will be no chance of recovery offered by the operation, as the inflammation will become more extensive the longer it continues, and, too, there will be so great a quantity of septic germs absorbed into the system that death will result from septicemia, even though the local inflammation should be remedied by a late operation. —*St. Louis Medical and Surgical Journal.*

